# Anti-inflammatory potential of Empagliflozin

**DOI:** 10.1007/s10787-021-00797-9

**Published:** 2021-03-17

**Authors:** Markus Pirklbauer

**Affiliations:** Department of Internal Medicine IV-Nephrology and Hypertension, Medical University Innsbruck, Anichstrasse 35, 6020 Innsbruck, Austria

**Keywords:** Empagliflozin, SGLT2 inhibition, Anti-inflammatory potential, Nephroprotection, Human proximal tubulus

Dear Editor

A recent article by Maayah and coworkers elegantly described a survival benefit with the use of the SGLT2 inhibitor Empagliflozin (Empa) in a murine model of LPS-induced septic shock (Maayah et al. 2020). The authors present evidence that the protective effect of Empa is at least partially mediated by a suppression of LPS‑induced renal and systemic inflammation and a reduction of LPS-induced acute kidney injury (AKI) (Maayah et al. 2020). SGLT2 inhibitors (SGLT2i) were developed as anti-glycemic drugs, attenuating renal glucose reabsorption by inhibiting the proximal tubular sodium/glucose co-transporter 2, thereby increasing urinary glucose excretion and lowering hyperglycemia (Vallon and Thomson 2020). HbA1c levels decrease by 0.5–0.7% on average with the use of SGLT2i. Large clinical trials, however, demonstrated SGLT2i-mediated renoprotection in type II diabetic patients with and without chronic kidney disease (CKD) irrespective of glycemic effects (Górriz et al. 2020). Moreover, the DAPA-CKD trial recently demonstrated SGLT2i-mediated nephroprotection in diabetic and non-diabetic CKD patients (Heerspink et al. 2020). Renal benefit has been primarily attributed to metabolic and hemodynamic drug effects, including lowering of systemic blood pressure, glomerular hyperfiltration, body weight, and plasma volume as well as reducing renal hypoxia. Direct anti-fibrotic mechanisms have also been demonstrated (Pirklbauer et al. 2019); however, underlying molecular mechanisms of SGLT2i are still incompletely understood.

Systemic and renal inflammation is causally involved in the initiation and progression of diabetic and non-diabetic kidney disease (Anders et al. 2018; Lv et al. 2018; Tesch 2017). Systemic levels of inflammatory markers (e.g., IL6, TNF-alpha, fibrinogen, and CRP) were found to be elevated in diabetic CKD patients and associated with renal damage (Festa et al. 2000; Giunti et al. 2010). During the initial CKD pathogenesis, proximal tubular cells start to proliferate, generate reactive oxygen species, and secrete pro-inflammatory cytokines. The latter promote the recruitment and activation of inflammatory cells, mostly mononuclear cells, to the glomerular and tubulo-interstitial compartment (Chevalier 2016; Vallon 2011; Vallon and Thomson 2020). Upon inflammosome activation, the recruited inflammatory cells release IL-1β which further aggravates renal tissue inflammation and promotes kidney damage (Anders 2016). In this regard, pharmacologic inhibition of IL-1β has been demonstrated to reduce the expression of renal damage markers and attenuate eGFR decline in murine CKD models (Shahzad et al. 2015; Orellana et al. 2017; Lei et al. 2019), as well as to reduce systemic inflammation in diabetic patients (Larsen et al. 2007).

Evidence for anti-inflammatory potential of SGLT2i (e.g., inhibition of inflammosome activation and reduced expression of inflammatory markers) has been previously demonstrated in diabetic animal models, and is associated with decreased glomerular and tubulo-interstitial damage (Benetti et al. 2016; Gembardt et al. 2014; Wang et al. 2017). Furthermore, SGLT2i attenuated high glucose-induced generation of reactive oxygen species and expression of pro-inflammatory markers (e.g., Toll-like receptor-4, NF-κB, collagen IV, and IL-6) in human proximal tubular cells (HPTC) (Ishibashi et al. 2016; Panchapakesan et al. 2013). In diabetic patients, SGLT2i were found to reduce systemic levels of pro-inflammatory (e.g., IL-6 and TNF-α) and pro-fibrotic (e.g., MMP7 and fibronectin 1) biomarkers (Heerspink et al. 2019) and to reduce urinary IL-6 concentration (Dekkers et al. 2018).

These SGLT2i-mediated anti-inflammatory effects, however, have not been shown under normoglycemic conditions until recently. In a non-diabetic rodent heart failure model, Byrne et al. demonstrated that Empa-mediated improvement of cardiac outcome is related to a reduced activation of cardiac inflammation (Byrne et al. 2020). In our own lab, we investigated the interference of Empa with tubular inflammatory response under normoglycemic conditions in two independent HPTC lines, namely HK-2 and RPTEC/TERT1 (Pirklbauer et al. 2020). At the human tubular transcriptome level, only 259 out of > 30.000 genes were uniformly upregulated by the pro-inflammatory mediator IL-1β (10 ng/ml), but downregulated by Empa (500 nM) in both cell lines. Functional annotation of these genes using pathway enrichment analysis (DAVID bioinformatics database) revealed that “cellular response to LPS” was among the highest annotated pathway cluster. This finding corroborates the work by Maayah et al. describing an Empa-mediated attenuation of LPS-induced systemic and renal inflammation in normoglycemic mice (Maayah et al. 2020). Basal and IL-1β-mediated expression of MCP-1/CCL2, a cytokine that is involved in early “inflammatory” pathogenesis of diabetic and non-diabetic kidney disease as well as cellular response to LPS (Kitagawa et al. 2004), was inhibited by Empa at the mRNA and protein level in both HPTC lines (Pirklbauer et al. 2020). Tubular MCP-1/CCL2 expression is increased both in experimental and human DKD (Chow et al. 2006; Mezzano et al. 2003) and elevated urinary concentrations are independently associated with higher eGFR decline in diabetic CKD patients (Tam et al. 2009; Satirapoj et al. 2018). Genetic knockdown and pharmacologic inhibition of MCP-1/CCL2 already demonstrated to attenuate inflammation, tubulus atrophy, albuminuria, and progressive fibrosis in several animal models of diabetic and non-diabetic kidney disease (Boels et al. 2017; Chow et al. 2006; Giunti et al. 2010; Kitagawa et al. 2004; Tarabra et al. 2009). Moreover, the MCP-1/CCL2 inhibitor Emapticap pegaol attenuated albuminuria in a phase II clinical trial among type II diabetic patients (Menne et al. 2003).

Based on the aforementioned central role of systemic and renal tissue inflammation during development and progression of CKD, it is tempting to speculate that anti-inflammatory effects of SGLT2i at least partially contribute to the nephroprotective effect observed in large clinical trials among diabetic and non-diabetic patients. However, in the absence of human data demonstrating anti-inflammatory SGLT2i effects on renal tissue level, kidney biopsy studies involving both SGLT2i-naïve and SGLT2i-treated patients are mandatory to confirm a potential association between anti-inflammatory and nephroprotective effects of SGLT2i.

The positive risk/benefit ratio found for SGLT2i use among non-diabetic CKD and heart failure patients in the DAPA-CKD (Heerspink et al. 2020) and DAPA-HF (McMurray et al. 2019) trials, respectively, might allow to test SGLT2i in the management of other non-diabetic kidney diseases. Based on the observed reduction of AKI in Empa-treated septic mice, Maayah et al. already suggested to study Empa treatment among septic patients requiring intensive-care therapy (Maayah et al. 2020). However, as these critically ill patients might be especially prone to SGLT2i-related severe adverse events, such as metabolic acidosis or life-threatening infectious complications, this approach must be critically reviewed. Immune-mediated glomerulonephritis, such as IgA nephropathy, or drug-related acute interstitial nephritis (AIN) that primarily involve “sterile” inflammation might be promising indications for future clinical trials assessing the renal benefit of SGLT2i. Most interestingly, IgA nephropathy was highly represented among those non-diabetic CKD patients that showed SGLT2i-related renal benefit in the DAPA-CKD trial. Nevertheless, cautious monitoring of SGLT2i-related side-effects, especially urogenital tract infections, is mandatory in future clinical trials.

## Data Availability

Not applicable.
